# Comprehensive geriatric assessment for people with both COPD and frailty starting pulmonary rehabilitation: a mixed-methods feasibility trial

**DOI:** 10.1183/23120541.00774-2023

**Published:** 2024-07-29

**Authors:** Lisa Jane Brighton, Catherine J. Evans, Morag Farquhar, Katherine Bristowe, Aleksandra Kata, Jade Higman, Margaret Ogden, Claire Nolan, Deokhee Yi, Wei Gao, Maria Koulopoulou, Sharmeen Hasan, Karen Ingram, Stuart Clarke, Kishan R. Parmar, Eleni Baldwin, Claire J. Steves, William D-C. Man, Matthew Maddocks

**Affiliations:** 1 King's College London, Cicely Saunders Institute of Palliative Care, Policy and Rehabilitation, London, UK; 2 King's College London, Department of Psychology, London, UK; 3 Sussex Community NHS Foundation Trust, Brighton General Hospital, Brighton, UK; 4 University of East Anglia, School of Health Sciences, Norwich, UK; 5 King's College London, Clinical Trials Unit, London, UK; 6 King's College London, Cicely Saunders Institute Public Involvement Group, London, UK; 7 Harefield Respiratory Research Group, Royal Brompton and Harefield Hospitals, Guy's and St Thomas’ NHS Foundation Trust, London, UK; 8 Brunel University London, College of Health, Medicine and Life Sciences, London, UK; 9 King's College Hospital NHS Foundation Trust, Pulmonary Rehabilitation, London, UK; 10 King's College Hospital NHS Foundation Trust, Department of Clinical Gerontology, London, UK; 11 Harefield Pulmonary Rehabilitation, Heart Lung and Critical Care Group, Guy's and St Thomas’ NHS Foundation Trust, London, UK; 12 Department of Geriatric Medicine, The Hillingdon Hospitals NHS Foundation Trust, Uxbridge, UK; 13 King's College London, Department of Twin Research and Genetic Epidemiology, London, UK; 14 Guys and St. Thomas’ NHS Foundation Trust, Department of Ageing and Health, London, UK; 15 Imperial College, National Heart and Lung Institute, London, UK; 16 King's College London, Faculty of Life Sciences and Medicine, London, UK

## Abstract

**Introduction:**

Many people with COPD experience frailty. Frailty increases risk of poor health outcomes, including non-completion of pulmonary rehabilitation. Integrated approaches to support people with COPD and frailty throughout and following rehabilitation are indicated. The aim of the present study was to determine the feasibility of conducting a randomised controlled trial of integrating comprehensive geriatric assessment (CGA) for people with COPD and frailty starting pulmonary rehabilitation.

**Methods:**

A multicentre mixed-methods randomised controlled feasibility trial (“Breathe Plus”; ISRCTN13051922) was carried out. People with COPD, aged ≥50 years, Clinical Frailty Scale ≥5 and referred for pulmonary rehabilitation were randomised 1:1 to usual pulmonary rehabilitation, or pulmonary rehabilitation plus CGA. Remote intervention delivery was used during COVID-19 restrictions. Outcomes (physical, psychosocial, service use) were measured at baseline, 90 and 180 days, alongside process data and qualitative interviews.

**Results:**

Recruitment stopped at 31 participants (mean±sd age 72.4±10.1 years, 68% Medical Research Council Dyspnoea Scale 4–5), due to COVID-19-related disruptions. Recruitment (46% eligible recruited) and retention (87% at 90- and 180-day follow-up) were acceptable. CGAs occurred on average 60.5 days post-randomisation (range 8–129) and prompted 46 individual care recommendations (median 3 per participant, range 0–12), 65% of which were implemented during follow-up. The most common domains addressed during CGA were nutrition and cardiovascular health. Participants valued the holistic approach of CGA but questioned the optimal time to introduce it.

**Conclusion:**

Integrating CGA alongside pulmonary rehabilitation is feasible and identifies unmet multidimensional need in people with COPD and frailty. Given challenges around timing and inclusivity, the integration of geriatric and respiratory care should not be limited to rehabilitation services.

## Shareable abstract

**Comprehensive geriatric assessment is feasible and acceptable for people with both COPD and frailty attending pulmonary rehabilitation; however, integrated geriatric and respiratory care should not be limited to rehabilitation services.** https://bit.ly/3U4lga3

## Introduction

Frailty is a multidimensional syndrome characterised by decreased reserve and diminished resistance to stressors [1], and affects one in five people with COPD [2]. People with COPD and frailty experience accumulating health problems and loss across physical, psychological and social domains [3]. This population is at increased risk of poorer health and function [4–6], as well as higher risk of hospitalisation [4] and mortality [4, 5], compared to those with COPD without frailty.

People with COPD and frailty face a “frailty rehabilitation paradox” [7]. Pulmonary rehabilitation is effective in improving health outcomes [6, 8] and reversing the physical elements of frailty [6, 9]. However, people with COPD and frailty encounter challenges to starting and completing outpatient programmes [3, 6]. Outpatient pulmonary rehabilitation typically comprises twice-weekly, supervised exercise sessions (involving progressive resistance and aerobic training based on individualised prescriptions) over 6–12 weeks, plus education to support self-management [10]. Development and testing of adapted approaches that better suit their decreasing reserves, multidimensional loss and changeable health are required to support engagement and improve outcomes.

Incorporating comprehensive geriatric assessment (CGA) alongside pulmonary rehabilitation may be a promising approach to support people with COPD and frailty to better engage with this service and experience improved health outcomes [3, 11]. CGA is a process of care that incorporates a multidimensional review of a person's medical, psychological, functional and social capability, in order to develop individual recommendations and a care plan [12]. In frail older adults, coordinated care based on CGA recommendations can improve quality of life and function, reduce hospital admissions [13], and reduce functional dependency and mortality [14, 15]. When introduced alongside other treatments such as surgery and chemotherapy, CGAs may increase tolerance, completion and outcomes [16, 17]. Recent work in inpatient respiratory rehabilitation incorporating a CGA-directed approach showed improved disease-specific health status and reduced exacerbations [18]. However, pulmonary rehabilitation rarely incorporates specialist geriatric expertise [19].

This study aimed to determine the feasibility of conducting a randomised controlled trial of an integrated CGA for people with COPD and frailty starting pulmonary rehabilitation. Objectives related to trial feasibility included: estimating success of recruitment and retention, estimating risk of contamination and unplanned unblinding, and exploring appropriateness and acceptability of the proposed outcome measures. Objectives related to intervention feasibility included exploring the acceptability of the CGA to participants and staff, and defining the content and understanding fidelity (including how the CGA differs from, and impacts on, usual care).

## Methods

### Design

A multicentre mixed-method randomised controlled feasibility trial was carried out. Full methods have been published previously [20] and are summarised below. The trial was prospectively registered (ISRCTN13051922).

### Setting

Participants were recruited from two outpatient pulmonary rehabilitation services in London, UK. An additional planned site withdrew due to service changes during the COVID-19 pandemic. The research team initially collected data at participants’ homes and then *via* telephone due to pandemic-related restrictions.

### Participants and recruitment

People aged ≥50 years, living with a physician diagnosis of COPD (in line with Global initiative for Chronic Obstructive Lung Disease (GOLD) criteria [21]), and scoring ≥5 on the Rockwood Clinical Frailty Scale [22], were eligible. All participants had been referred for outpatient pulmonary rehabilitation, in line with British Thoracic Society guidance [10]: able to walk at least 5 m, experiencing functional impairment due to breathlessness, no previous supervised pulmonary rehabilitation in the previous 12 months, and moderate-intensity exercise deemed safe. Specific comorbidities did not preclude eligibility for rehabilitation, so long as the above criteria were met. People without mental capacity to provide informed consent were excluded from the study; this was determined by the referring clinician or researcher, based on the person's ability to understand, retain, weigh up and communicate relevant information [23]. People unable to communicate in English (and no interpreters available to enable this) or receiving care from a geriatrician in the previous or upcoming month were also excluded from the study.

Participants were sampled consecutively from pre-pulmonary rehabilitation assessments. Pulmonary rehabilitation professionals screened people with COPD for eligibility and referred interested eligible participants to the research team.

### Interventions

#### Usual care

All usual care contacts were permitted in both trial arms and were recorded using the Client Service Receipt Inventory [24]. All participants were scheduled for pulmonary rehabilitation at baseline. This typically comprised twice-weekly supervised outpatient exercise sessions, plus education, for 6 weeks (site A) or 8 weeks (site B). Some attended remote-facilitated rehabilitation due to COVID-19 restrictions.

#### Comprehensive geriatric assessment

In addition to usual care, the intervention group were referred for a CGA as soon as possible following completion of baseline measures. Our intention was for the CGA to occur prior to starting pulmonary rehabilitation, to add value to their rehabilitation while also addressing frailty and its consequences.

CGA involves a holistic assessment, development of a tailored care plan, and follow-up as required [25]. The CGA was led by a geriatrician within the multidisciplinary geriatrics team, following their usual local clinic proformas and practice. Initial appointments typically lasted about 1 h, and included a full medical assessment and history, and typically a review of functional and psychosocial issues, management of geriatric syndromes (*e.g.* frailty, falls, sarcopenia, incontinence, malnutrition, sensory impairment) and advance care planning, as relevant for the person. A resulting individualised care plan was created in collaboration with the participant. The plan was then shared with the participant and relevant healthcare professionals for actioning. In all cases, tailoring of the CGA, subsequent follow-up and decision to discharge was led by the geriatrician, but with access to the wider multidisciplinary team (*e.g.* nursing, physiotherapy, occupational therapy) if required. Appointments were planned to be in-person in an outpatient clinic, but some were delivered by telephone due to COVID-19 restrictions. When delivered remotely, the same proformas were followed in terms of domains covered. The teams followed established local protocols to ensure safety, including arranging for further in-person follow-up as required.

### Feasibility outcomes and progression criteria

*A priori* progression criteria for each feasibility outcome were described previously [20]. An additional feasibility outcome around describing the theoretical underpinning of the intervention will be reported separately.

The trial feasibility outcomes were as follows:

- Identification and recruitment of eligible participants
- Participant retention at follow-up
- Contamination of the control group
- Success of data collector blinding
- Acceptability of outcome measures and their timing

Intervention feasibility outcomes were:

- Acceptability of the intervention to participants and staff
- Fidelity of delivery of recommendations from the CGA
- Defining what and how many recommendations are made in the CGA
- Defining what usual care comprises

### Data collection

We collected the following:

- Process data: implementation of the CGA (timing, participation, mode of delivery, care recommendations), pulmonary rehabilitation participation (mode, proportion of sessions attended, reasons for disruptions) and trial processes (screening, recruitment, participation and missing data).
- Participant characteristics and clinical outcomes: baseline demographic characteristics, health and function, and socioeconomic factors. Clinical outcome measures collected at baseline, 90 and 180 days included: the Short Physical Performance Battery (SPPB) [26], Chronic Respiratory Questionnaire (CRQ) [27], Manchester Respiratory Activities of Daily Living questionnaire [28], Euro-Qol 5D-5L, Hospital Anxiety and Depression Scale [29] and the De Jong Gierveld Loneliness Scale [30].
- Participant and staff experiences: nested semi-structured qualitative interviews were conducted with a sub-sample of intervention group participants and staff to address feasibility objectives around intervention acceptability, and acceptability of outcomes and their timing (topic guides in supplementary tables S1 and S2). Participants were purposively sampled by site and intervention fidelity (diverse proportions of completed CGA recommendations) with consideration of diversity in terms of living status, outcomes and questionnaire completion where possible. Staff were purposively sampled by site and role (geriatrics *versus* pulmonary rehabilitation). All interviews took place remotely *via* telephone or videocall.

### Sample size

We intended to recruit 60 participants (30 per study group) to achieve the desired level of precision in the quantitative feasibility outcomes [31].

### Randomisation and allocation concealment

Participants were randomly allocated 1:1 to the intervention or control group. We used minimisation with a random element to help balance the groups in terms of study site (A/B), Medical Research Council Dyspnoea Scale (2–3/4–5), exacerbations in the past year (<2/≥2) and living alone status (Yes/No) [32]. Randomisation was conducted using an independent web-based randomisation system, *via* King's Clinical Trials Unit.

### Blinding

The researcher collecting follow-up data was blinded at 90-day follow-up, but unblinded before 180-day follow-up to facilitate invitation to qualitative interviews. It was not possible to blind participants or intervention providers.

### Analysis

Feasibility outcomes are presented using a descriptive intention-to-treat analysis. Data are expressed as proportions and corresponding 95% confidence intervals, or as mean±sd and median (range), depending on the data distribution. Baseline characteristics and clinical outcome data are summarised using descriptive statistics.

Qualitative data were analysed using framework analysis [33], led by an experienced qualitative researcher (L.J. Brighton) with interpretative input from the wider research team, including people with relevant lived experience. The frameworks drew deductively on our preliminary intervention theory and the theoretical framework of acceptability [34], as well as being open to inductively generated participant-raised issues.

Decision to progress to a full trial was determined based on feasibility trial results meeting pre-defined criteria, assessed by the trial team alongside context provided by the qualitative data [35].

### Patient and public involvement

Public involvement members affected by COPD and frailty informed the development and conduct of this feasibility trial. This included both public members with previous research involvement experience, and some who were new to this process. They contributed to design of recruitment processes, participant materials and outcome measurement, interview topics and prompts, troubleshooting challenges of remote data collection, and interpretation and reporting of findings. Reflections on public involvement in the trial can be found in supplementary table S3.

### Ethics

This study was approved by the London City and East Research Ethics Committee (Ref. 19/LO/1402). All participants provided informed consent.

### Protocol version and amendments

This paper reflects version 3.0 of the study protocol dated 25 June 2020; no further substantial amendments have been made since protocol publication.

## Results

### Recruitment and retention

Participants were recruited between November 2019 and July 2022, with several interruptions due to the COVID-19 pandemic. In total, recruitment was open 16 months at one site, and 11 months at the other.

**FIGURE 1:**
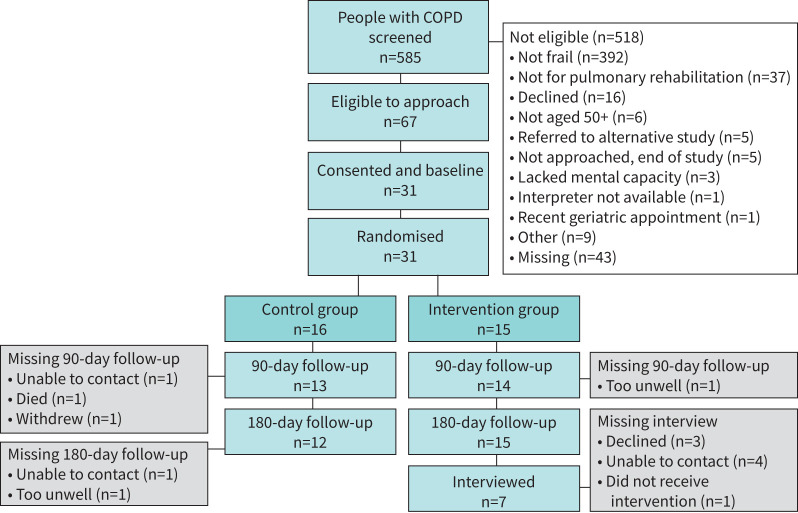
Participant flow through the feasibility trial.

Of 585 people with COPD screened, 67 (11%) were eligible, and 31 (46%) were recruited to the study. All 31 participants were randomised: 16 to the control group and 15 to the intervention group. 27 participants (87%) completed the 90-day follow-up and the 180-day follow-up (figure 1). Baseline participant characteristics are shown in table 1.

**TABLE 1:** Feasibility trial participant characteristics (n=31)

Characteristic	Total	Usual care	Usual care + CGA
**Participants n**	31	16	15
**Age years**	72 (66–82)	71 (67–82)	74 (62–82)
**Female sex^#^**	19 (61.3)	9 (56.3)	10 (66.7)
**Ethnicity** ^¶^			
** **Asian, black or mixed	4 (13.0)	2 (12.5)	2 (13.3)
** **White	26 (83.9)	13 (81.3)	13 (86.7)
** **Other	1 (3.2)	1 (6.3)	-
**English indices of multiple deprivation (decile)**	4 (2–6)	5 (2–6)	4 (2–5)
**Lives alone**	13 (41.9)	6 (37.5)	7 (46.7)
**Housing** ^¶^			
** **Homeowner (outright or with mortgage)	15 (48.4)	10 (62.5)	5 (33.3)
** **Renting privately	6 (19.3)	3 (18.8)	3 (20)
** **Council, local authority or housing association house	10 (32.3)	3 (18.8)	7 (46.7)
**Education** ^¶^			
** **Left school aged ≤15 years	18 (58.1)	8 (50)	10 (66.7)
** **Left school aged 16–19 years	7 (22.6)	3 (18.8)	4 (26.7)
** **Post-secondary or university qualification	6 (19.3)	5 (31.3)	1 (6.7)
**Has an unpaid/family carer**	24 (77.4)	13 (81.3)	11 (73.3)
**Provides unpaid/family care for one or more adults**	7 (22.6)	4 (25)	3 (20)
**FEV_1_ % predicted**	51 (36–71)	51 (32–74)	52 (38–70)
**GOLD grade** ^¶^			
** **1–2	14 (45.2)	8 (50)	6 (40)
** **3–4	13 (41.9)	7 (43.8)	6 (40)
** **Missing	4 (12.9)	1 (6.3)	3 (20)
**GOLD grade 2023**			
** **B	13 (41.9)	6 (37.5)	7 (46.7)
** **E	18 (58.1)	10 (62.5)	8 (53.3)
**Exacerbations of COPD in past year**			
** **0	9 (29.0)	4 (25)	5 (33.3)
** **1	8 (25.8)	4 (25)	4 (26.7)
** **2+	14 (45.2)	8 (50)	6 (40)
**Medical Research Council Dyspnoea**			
** **2–3	10 (32.3)	5 (31.3)	5 (33.3)
** **4–5	21 (67.7)	11 (68.8)	10 (66.7)
**Clinical Frailty Scale score**			
** **5	26 (83.9)	12 (75)	14 (93.3)
** **6	5 (16.1)	4 (25)	1 (6.7)
**Number of comorbidities**	4 (3–6)	4 (3–7)	4 (3–6)
**Study site**			
** **Site A	20 (64.5)	10 (62.5)	10 (66.7)
** **Site B	11 (35.5)	6 (37.5)	5 (33.3)
**Smoking status**			
** **Current smoker	8 (25.8)	5 (31.3)	3 (20)
** **Ex-smoker	21 (67.7)	10 (62.5)	11 (73.3)
** **Never-smoker	2 (6.5)	1 (6.3)	1 (6.7)
**Incremental Shuttle Walk Test m^+^**	125 (80–218)	80 (70–228)	155 (98–218)

Data are presented as median (IQR) or n (%). FEV_1_: forced expiratory volume in 1 s; GOLD: Global Initiative for Chronic Obstructive Lung Disease. ^#^: self-described gender matched sex at birth in all cases; ^¶^: categories combined to preserve anonymity; ^+^: n=20 (n=8 usual care, n=12 usual care + CGA) due to changes in practice during COVID-19 restrictions.

### Data collection

Median missing data across measures over all timepoints was 5.9% (range 0–87.1%). The highest proportions of missing data were for the SPPB (87.1% administrations), because it could not be administered by phone during COVID-19 restrictions, and the CRQ Dyspnoea scale (14.1% administrations), typically because important activities selected at baseline had become non-applicable at follow-up due to functional decline or COVID-19 restrictions. Remaining missing data generally resulted from participants feeling too fatigued or unwell to complete the entire questionnaire booklet. Missing data are shown in supplementary table S4. Median scores on the clinical outcome measures across groups and timepoints are shown in supplementary table S5.

Qualitatively, participants described the outcome measures as easy to complete and not burdensome. They found some questions challenging in deciding their response, and one person noted it required them to consider their health in detail when they typically try not to. However, participants generally reported enjoying sharing experiences and reflecting on changes, and the questions were deemed to cover a good spectrum of relevant areas.

### Unblinding and contamination

Unplanned unblinding occurred in two cases (6%), where intervention participants mentioned the geriatrician during the follow-up data collection. There were no instances of participants in the control group receiving the CGA intervention.

### Usual care

Most participants (22 out of 31) were assigned to in-person centre-based pulmonary rehabilitation, five to a remote alternative (phone or video), and four began in-person and later switched to remote delivery. The median proportion of scheduled pulmonary rehabilitation sessions attended was 35.7% (50.0% in the intervention group and 28.1% in the control group); disruptions were generally attributed to COVID-19 restrictions and illness.

Complete service use data were available for 24 out of 31 participants. Alongside pulmonary rehabilitation, the most common usual care contacts during the trial were with a general practitioner by phone (n=17) and in-person (n=11), a practice nurse (n=11) and hospital-based specialist outpatient clinics (n=12). Most participants also reported some level of unpaid/family carer support during the trial: commonly help outside the home (n=21) and at home (n=14), plus time spent “on call” (n=10). Full data on usual care contacts are shown in supplementary table S6.

### Intervention fidelity

14 of the 15 intervention group participants received a CGA, a median of 60.5 days (range 8–129) following randomisation. The participant who did not attend the intervention reported not receiving the appointment invitation. Three participants received their CGA before pulmonary rehabilitation, three received it during their pulmonary rehabilitation and four received the CGA after their pulmonary rehabilitation (four did not attend pulmonary rehabilitation).

Participants receiving the intervention had a median of 1 appointment (range 1–2) with the geriatrician during the study period. Eight participants had in-person appointments, five had telephone appointments, and one received a telephone CGA prompting an in-person follow-up.

CGAs prompted 46 individual care recommendations (median 3 per participant, range 0–12). Table 2 shows the domains and types of care recommendations, excluding five recommendations for repeat review by the geriatrics team. Most common recommendations related to changing non-respiratory medications (including starting, adjusting or stopping medications) and onward referral, and the most common domains addressed included nutrition and cardiovascular health. None of the recommendations related to their pulmonary rehabilitation. Of these 46 recommendations, 30 (65%) were completed by the end of study follow-up. Reasons for non-completion included COVID-19 disruptions (n=5) and participant choice (n=1), though for 10 recommendations the reasons for non-completion were unknown.

**TABLE 2:** Recommendations from the comprehensive geriatric assessment (excluding repeat reviews; n=41)

Recommendation domain	Recommendation type					Total domain	Illustrative examples of recommendations^#^
	Referrals	Non-respiratory medication	Self-management	Investigation	Supplements		
**Nutrition**	1	-	2	-	4	7	Participant to try more [no alcohol days] in January
**Cardiovascular**	2	3	-	1	-	6	GP to consider referring for repeat ECHO
**Gastroenterology**	-	4	-	-	-	4	Participant to stop oral antibiotics
**Societal participation**	1	-	3	-	-	4	Start diary of physical activity
**Cognitive**	1	-	-	2	-	3	Referral to memory clinic
**Neurological**	2	-	-	1	-	3	Referral to consultant neuro-geriatrician
**Psychological**	2	1	-	-	-	3	Referral to hospice bereavement counselling service
**Respiratory**	3	-	-	-	-	3	Referral to smoking cessation
**Environment**	1	-	1	-	-	2	Pursue help from gardener
**Pain**	-	2	-	-	-	2	Change in pain medication due to side-effects
**Metabolic**	-	1	-	-	-	1	Consider oral hypoglycaemics
**Musculoskeletal**	-	1	-	-	-	1	Consider bone protection due to steroid use
**Urinary**	-	1	-	-	-	1	Prescription to address urinary tract symptoms
**Ear and labyrinth**	1	-	-	-	-	1	Urgent Ear, Nose & Throat referral following head CT
**Total type**	14	13	6	4	4	41	

^#^: paraphrased to preserve anonymity.

### Intervention acceptability

Seven intervention group participants were interviewed, representing diversity in study site, proportion in CGA actions completed, living alone *versus* with others and changes in CRQ scores (supplementary table S7). All participants had completed all study questionnaires, and none opted to include unpaid/family carers in the interview. Five professionals involved in the trial were also interviewed, representing diversity in study site and discipline (supplementary table S8). Illustrative quotes relating to domains of acceptability are shown in table 3.

**TABLE 3:** Illustrative quotes relating to domains of acceptability

Domain: Definition	Participants^#^	Professionals^¶^
**Affective attitude:** Feelings about intervention	“…it gives you the sense that you are somebody and not just a number.” (Ivy) “It's like an MOT of your body.” (Janice)	“I loved the fact that for some of these patients, they would get more support, more investigation, a more holistic, kind of, review.” (S03)
**Burden:** Perceived effort	“That sort of thing doesn't bother me” … “If you've got another appointment, then you've got another appointment.” (Susan)	“And patients have got lots and lots of appointments. I completely understand that this is just an additional thing on top of that, but it all depends.” (S04)
**Ethicality:** Alignment with values	“[the doctor] gave me the feeling that they had all the time in the world to explain things to me. And in this day and age that's very valuable.” (Ivy)	“…patients who are referred to pulmonary rehab, but they cannot attend because of memory issues or…poor balance. What are we doing for these patients?”(S02)
**Intervention coherence:** Understanding how it works	“…when you've got one particular issue, it can have a great impact on another issue, and if somebody says, ‘Yes, I get that, but that's not my side of things, so you'll have to see somebody else about that’ – So it was really, really good to have somebody who would take an overall view.” (Claire)	“So, I think that looking at this in respiratory medicine and pulmonary rehab is absolutely valid because actually that's how I saw us doing work when I first started in geriatric medicine.” (S01)
**Opportunity costs:** What is lost by taking part	“To be honest, it had crossed my mind”…“I was scared it was going to be [diagnosis].” (Susan)	“…we have to sort of respect sort of erm any potential issues that we highlight that the patient may not have specifically come to clinic for and may not want any ongoing interventions for.” (S05)
**Perceived effectiveness:** Whether it's seen to achieve its purpose	“Very helpful y'know, yeah. Because obviously I wouldn't have known if I had a vitamin D… deficiency, and obviously [the doctor] gave me the tablets for my prostate and it's all fine. So it's good.” (David)	“And you know you'll identify things that you can potentially put an intervention in, and improve their quality of life. So I guess coinciding that with their pulmonary rehab sort of overall you hope that you're able to add some value to their ongoing management.” (S05)
**Self-efficacy:** Confidence to perform behaviour(s) required	“I felt that the suggestions were achievable, and it was, just as I say, ‘Well, just take small steps to begin with and build up from there.’ And I do things now, as I say, that make sure I keep abreast of things.” (Claire)	“There are things that we can do to improve people's input, I'd say, at any level of clinical frailty.” (S01)

Note: participant names are pseudonyms. ^#^: n=7; ^¶^: n=5.

**TABLE 4:** Results relating to pre-specified feasibility outcomes and progression criteria

Feasibility outcomes	Progression outcome	Supporting data
**Trial feasibility**		
** **Identification and recruitment of eligible participants	• Amber: 10–19% screened eligible	11% (95% CI 9–14%) screened eligible
	• Amber: 40–59% eligible recruited	46% (95% CI 34–59%) eligible recruited
** **Participant retention at follow-up	• Green: ≥75 retained at 90 days	87% (95% CI 70–96%) retained at 90 days
	• Green: ≥60% retained at 180 days	87% (95% CI 70–96%) retained at 180 days
** **Contamination of the control group	• Green: ≤10% receive a CGA within usual care	0% (95% CI 0–21%) contamination in control group
** **Success of data collector blinding	• Green: blinding maintained for ≥85% of participants	94% (95% CI 79–99%) maintained, with changes to unblinding procedure to facilitate earlier invite to interviews
** **Acceptability of outcome measures and their timing	• Amber: missing data of 11–25% for each measure	Median missing data 5.9%. Green criteria met for all measures except CRQ Dyspnoea (amber) and SBBP (red, due to COVID-19 restrictions). Amber criteria agreed to reflect remediable issues
	• Acceptable to participants	Acceptable to participants
**Intervention feasibility**		
** **Acceptability of the intervention to participants and staff^#^	• Generally acceptable	Acceptable and positive consultation experience, variable perceived benefits
** **Fidelity of delivery of recommendations from the CGA^#^	• Amber: 79–50% implemented	65% (95% CI 50–79%) recommendations implemented. 10% were not completed due to COVID-19 disruptions
** **Defining what and how many recommendations are made in the CGA	• Described	Median 3 (range 0–12) recommendations per participant. Most common related to onward referral and non-respiratory medication
** **Defining what usual care comprises	• Described	Common contacts include GP, practice nurse and specialist outpatients, plus unpaid/family carer support. Median 35.7% of pulmonary rehabilitation sessions attended

Traffic-light progression criteria [35] – green: likely no concerning issues; amber: potentially remediable issues; red: potentially intractable issues. CGA: Comprehensive Geriatric Assessment; CRQ: Chronic Respiratory Questionnaire; SPPB: Short Physical Performance Battery. ^#^: primary focus.

### Participants

Participants often reported positive experiences of the intervention: they particularly appreciated the whole-person approach, the assessment's comprehensiveness and time taken, and the opportunity to share concerns with nothing being “out of remit”. Where applicable, they felt capable of completing the recommendations assigned to them. The extra appointment was not deemed too burdensome, so long as the resulting recommendations felt relevant and helpful. One participant, who received an in-person CGA, queried whether it could have been done by telephone to reduce travel, but acknowledged the physical tests they underwent would not have then been possible.

Following the CGA, some participants reported feeling supported and motivated in their self-management, reported improved mood through relieving concerns and noted the benefits of prompting further investigations. Others were unclear on the relevance to them and perceived little to no impact beyond the positive experience of the appointment. This was more common for participants where the CGA recommendations raised issues that they did not want to follow-up with (*e.g.* memory difficulties, addiction), or where the recommendations had not yet been actioned.

### Professionals

All professionals agreed on the potential for CGA to identify and address unmet needs. They felt that introducing CGA for this group addressed a gap in current practice, and that it could have further impact by extending reach to those with COPD and frailty unable to attend rehabilitation. Professionals also suggested that the approach could be strengthened by providing participants with more information about the role of CGA in advance, and by using a more integrated approach (for example, using multidisciplinary team meetings to discuss shared patients).

The pulmonary rehabilitation physiotherapists had conflicting views about the extent to which, if the CGA intervention had been received earlier (as intended), the CGA recommendations might have influenced pulmonary rehabilitation delivery. However, the challenges of timing delivery of the CGA between the rehabilitation assessment and start date were acknowledged, particularly given service metrics focused on shortening this gap. Alongside the CGA, professionals suggested there may be other ways to consider adapting pulmonary rehabilitation for this population, including more tailored education for people with frailty, closer supervision, addressing balance, adapting aerobic exercises and offering more home-based sessions.

### Adverse events

17 participants (55%) experienced adverse or serious adverse events during the trial (n=13 control and n=11 intervention). There were 10 adverse and three serious adverse events in the control group, and six adverse and five serious adverse events in the intervention group. Most serious adverse and adverse events were respiratory (*e.g.* exacerbations of COPD and related hospital admissions), followed by musculoskeletal issues (*e.g.* muscle weakness/pain), blood and lymphatic systems (*e.g.* anaemia) and psychiatric concerns (*e.g.* confusion, distress). No adverse events reported were deemed related to the intervention.

### Summary of feasibility outcomes

A summary of the findings in relation to the pre-determined feasibility outcomes and progression criteria are shown in table 4.

## Discussion

A randomised trial integrating CGA for people with COPD and frailty referred for pulmonary rehabilitation was acceptable to participants and technically feasible. However, an approach that integrates CGA into the wider respiratory care pathway (rather than timing alongside pulmonary rehabilitation) may be preferable. Trial recruitment and retention were generally successful and suggest measurement of the proposed clinical outcomes over 180 days would be possible. The data show that a CGA and resulting recommendations can be successfully delivered to this population, particularly when services have recovered from COVID-19 disruptions. CGA recommendations covered a variety of issues ranging from nutrition and cardiovascular issues to cognitive and psychological concerns, which had not been addressed through participants’ existing contacts with pulmonary rehabilitation, general practice and specialist teams. Participants particularly valued this holistic approach to their health.

This is the first randomised feasibility trial to integrate CGA alongside outpatient pulmonary rehabilitation. Our findings align with cohort studies of an inpatient geriatric rehabilitation approach “GR-COPD” in the Netherlands, which also found that CGA may have a role in identifying and addressing heterogeneous and multidimensional unmet needs of this population [18, 36]. Onward referral and adjusting non-respiratory mediation were common CGA outcomes in our study, highlighting the potential added value of geriatric expertise, which is particularly suited to working with people with multiple long-term conditions, addressing polypharmacy and taking a whole-person approach.

Despite the potential added value of CGA, our findings suggest the start of pulmonary rehabilitation may not be the optimal time to refer people with COPD and frailty for this combined intervention. It was proposed that timing the CGA close to the start of pulmonary rehabilitation attendance might support engagement in this service, similar to timely benefits of pre-surgery [16] or pre-chemotherapy [17] CGAs. However, our process data showed the median time between randomisation and receipt of CGA was around 2 months, limiting the potential impact on pulmonary rehabilitation attendance and completion. This was true even for participants recruited prior to COVID-19-related disruptions. Moreover, several people with COPD and frailty declined participation in the trial due to “too much going on”, including some who specifically said they would have been interested if it was not offered around the same time as their pulmonary rehabilitation. Our cohort also only included people with Clinical Frailty Scale scores as high as 6, likely reflecting the limited cohort of people with COPD and frailty able to participate in pulmonary rehabilitation. These findings substantiate the qualitative responses from professionals in our trial, who felt that the potential benefits of CGA could extend to those patients unable to attend pulmonary rehabilitation. Therefore, progression to a full trial of the current protocol is not recommended.

Our feasibility study included a smaller sample than intended due to COVID-19-related disruptions. Although feasibility studies of other respiratory rehabilitation models have similar samples sizes [37–40], the early stopping of recruitment reduces the level of precision in the quantitative feasibility outcomes. Our study was successful in adapting to a shifting context by incorporating remote methods of intervention delivery and outcome collection during the COVID-19 pandemic; adaptations which we have detailed comprehensively for transparency [41]. While this adds further complexity to our findings, it was still possible to meet our study aims; integration of both quantitative and qualitative data provided additional depth and context to support robust interpretation. This mixed-method approach was highly valuable as feasibility outcomes were generally positive, but unanticipated challenges outside of the pre-determined feasibility parameters were still encountered (*e.g.* intervention timing). While our sample includes good representation of people from a range of ages, disease severity and from areas of socioeconomic deprivation, additional efforts to ensure inclusion of more participants from minority ethnic groups would strengthen future work. While participants who had difficulties completing the questionnaires were not represented in our qualitative sample, we still captured many important areas of diversity (*e.g.* study site, intervention fidelity, staff discipline) that may impact acceptability. Our work did not explore the impact of this intervention on unpaid/family carers; this could strengthen a future trial.

Given the challenges around timing the CGA alongside pulmonary rehabilitation and the issues of equity of access raised by professionals in our study, alternative models of integrating CGA for people with COPD and frailty warrant exploration. Alternative strategies might include referral for CGA *via* primary care and outpatient respiratory clinics, and models incorporating multidisciplinary team meetings for shared patients. Similar models have been successful in integrating respiratory and palliative care [42, 43], and are being trialled for integrating geriatric care for people with HIV [44]. Alongside this, further attention will also be needed to address the “frailty rehabilitation paradox”. For example, a recent American Thoracic Society Workshop Report on rehabilitation for people with respiratory disease and frailty suggests that adapting exercise prescription and monitoring to suit high symptom burden and/or proactively planning for disruptions should be important considerations for rehabilitation delivery in this population [7]. Rigorous studies testing these adaptations and their impacts on engagement and outcomes in people with frailty are required.

In conclusion, a trial of integrating CGA in people with COPD and frailty is feasible, and the CGA can identify unmet needs in this group. Given challenges around timing the CGA process alongside pulmonary rehabilitation, models of integrating geriatric and respiratory care that are not limited to rehabilitation services may be more practical and inclusive. Direct alterations to support engagement of people with COPD and frailty in pulmonary rehabilitation services warrant further study.

## Supplementary material

10.1183/23120541.00774-2023.Supp1 **Please note:** supplementary material is not edited by the Editorial Office, and is uploaded as it has been supplied by the author.
Supplementary material 00774-2023.SUPPLEMENT
