# Supplementary material for: Comprehensive geriatric assessment for people with both COPD and frailty starting pulmonary rehabilitation: a mixed-methods feasibility trial
Source: ERJ Open Res. 2024 Jul 29;10(4):00774-2023. doi: 10.1183/23120541.00774-2023 (PMC11284595; doi:10.1183/23120541.00774-2023)
Supplement: Supplementary file 1 [file 00774-2023.SUPPLEMENT.pdf]

Supplementary Material

Comprehensive Geriatric Assessment for people with both COPD and frailty starting pulmonary rehabilitation: a mixed-methods feasibility trial

Lisa Jane Brighton, Catherine J Evans, Morag Farquhar, Katherine Bristowe, Aleksandra Kata, Jade Higman, Margaret Ogden, Claire Nolan, Deokhee Yi, Wei Gao, Maria Koulopoulou, Sharmeen Hasan, Karen Ingram, Stuart Clarke, Kishan Parmar, Eleni Baldwin, Claire J. Steves, William D-C Man, Matthew Maddocks

Contents

Supplementary Table S1: Topic guide for interviews with trial participants 2
Supplementary Table S2: Topic guide for interviews with professionals involved in the trial 3
Supplementary Table S3: Reflections on public involvement using the GRIPP short form checklist 4
Supplementary Table S4: Missing item(s) in each clinical outcome measure by timepoint 6
Supplementary Table S5: Scores on clinical outcome measures at each timepoint (median [IQR]) 7
Supplementary Table S6: Service use at baseline and during the trial 8

Supplementary Table S1: Topic guide for interviews with trial participants

Main questions	Prompts if needed
Intervention acceptability and how it might work	
**1. As part of the study you were scheduled for an additional appointment during ________. Can you tell me about how you were getting on at that time?**	What were you concerned about at that time?<br>What were your priorities at that time?<br>Who was supporting you at that time? (this might include health or social care professionals, or friends and family)
**2. You had an appointment scheduled on ________________ with Dr ________________ from the ___________________________. Can you please talk me through what happened?**	Was the appointment in person, or by phone?<br>(If applicable) How did you get to the appointment?<br>Did you need to ask for any special arrangements?<br>Did someone come with you for the appointment? Did they stay throughout?<br>What did you discuss / What did they suggest?<br>What happened afterwards?
**3. How did it feel to have this appointment?**<br><br>**4. How did you feel about what they suggested?**	What did you like about it, if anything?<br>What didn't you like about it, if anything?<br>(If applicable) What did [informal carer] think of the appointment?<br><br>How achievable did you think they were?
**5. How much effort was it to attend the appointment?**<br><br>**6. How much effort has it been to follow up with their suggestions?**	What challenges, if any, did you face in attending the appointment?<br><br>What challenges, if any, did you face in [suggested actions]?
**7. How helpful or not did you find the appointment?**<br><br>**8. How helpful or not did you find their suggestions?**	In what way(s)?<br>Did you notice anything change as a result of the appointment/their suggestions?<br>Did going to the appointment / their suggestions make anything worse?
**9. Why do you think that it was helpful/unhelpful?**	What was it about the appointment that was helpful/unhelpful?<br>Did you feel listened to?<br>What might have made it more helpful?<br>How did you find the location? How different do you think it might have been if it was in-person / by phone? Please tell me why.
Acceptability of the questionnaires and their timing	
**10. How did it feel to do the questionnaires?**	(If applicable) What about the physical tests we did?<br>(If applicable) How did you find doing the questionnaires by phone?
**11. How much effort was it to complete the questionnaires?**	How was the length of the questionnaires?<br>How was it completing them three times?
**12. How well did you think the questionnaire captured how you were feeling?**	Which felt the most relevant to you?<br>Which felt the least relevant to you?<br>Was there anything missing?

Supplementary Table S2: Topic guide for interviews with professionals involved in the trial

Main questions	Prompts if needed
Intervention acceptability	
**To begin with, please can you tell me about your role in the study?**	[tailor the follow-up questions based on type of involvement]<br><br>For CGA delivery:<br>- Can you please talk me through what a CGA looks like in your practice?<br>- How much of the intervention is standardised/tailored?<br>- Who contributes to this process?
**How did you find contributing to these parts of the study?**	Were there any negative or challenging aspects?<br>Were there any positive or helpful aspects?<br>How did you find (as applicable):<br>- Identifying participants (in-person vs virtual)<br>- Introducing the study (in-person vs virtual)<br>- Passing information to the researcher<br>- Scheduling people for a CGA<br>- Delivering the CGA (in-person vs virtual, timing)<br>- Following up with actions from the CGA<br><br>Were there any differences in how these processes were planned, and how they played out in practice?<br>What kinds of impacts, if any, did the Covid-19 pandemic have on these processes for you?<br>How noticeable, or not, was the integration between the services in your role?
**Do you have any reflections on how we can improve delivery of the study/intervention in the future?**	How burdensome or not were these processes for you?<br>Are there areas in which the research team might be able to provide more support / training / resources?
Intervention theory	
**What are your thoughts on referring people with COPD and frailty for a comprehensive geriatric assessment towards the start of pulmonary rehabilitation?**	How relevant (or not) do you think this approach is for this population?<br>What do you think the potential benefits are?<br>What do you think the potential challenges are?<br>What impacts might this approach have?<br>What other opportunities might there be for working together with geriatrics in pulmonary rehabilitation, if any?
[Show preliminary CGA data]<br>**Having looked at these preliminary findings, do you have any reflections you'd like to share?**	Do any of these preliminary findings surprise you?<br>Are any of the findings in line with what you expect?<br>What do you think are the key takeaways from this so far?<br>What are your thoughts on:<br>- Identifying the right people with COPD for a CGA<br>- When might be best to offer a CGA?<br>- How best to integrate respiratory and geriatric input?

Supplementary Table S3: Reflections on public involvement using the GRIPP short form checklist

1: Aim: Report the aim of Patient and Public Involvement (PPI) in the study

The aims of public involvement in this project were to improve the acceptability of the research processes and proposed intervention, broaden and enrich the interpretations of the findings, and improve the clarity and impact of how the findings are shared with others.

2: Methods: Provide a clear description of the methods used for PPI in the study

The researcher worked together with people with lived experience relevant to COPD and frailty (henceforth 'service user representatives'), with and without previous experience public involvement in research. This included:

- A small project-specific group, including members of the Harefield Breathing Group, who met periodically in-person and virtually, and kept in touch by phone and email in between.
- Members of the wider Cicely Saunders Institute public involvement group, who provided feedback during in-person and virtual 'Dragons' Den' workshop sessions
- Members of the Biomedical Research Council Respiratory Research group, who provided feedback in in-person and virtual workshops

Methods of involvement depended on the circumstance, but ranged from structured presentations with questions for feedback, to broader open discussion about the project components, and invitation to comment on specific documents. In all cases the input of public members was acknowledged and recorded in a public involvement log.

3: Study results: Report the results of PPI in the study, including both positive and negative outcomes

The project advisory public members came up with the name 'Breathe Plus' for the feasibility trial. Feedback on the trial design highlighted the importance of thinking about frailty in a holistic sense (including psychological aspects), contributing to thinking about the most appropriate screening measure. The researcher also revised the information materials in line with their suggestions to refer to just a 'comprehensive assessment' as participants would not identify with 'geriatric', and to remove reference to frailty as they felt it was not necessary and may put people off. They also helped the researcher provide clear information about how participants' data were handled, and what happened to control group participants in the trial. When recruiting participants within pulmonary rehabilitation assessments, they noted that people often leave with lots of written information, and so suggested the researcher include a postcard piece of study information as an alternative for those feeling overwhelmed. Their comments also helped improve the formatting and clarity of the questionnaires, and the comprehensiveness of the qualitative interview topic guides. When adapting the study to Covid-19, public members advised the researcher about the importance of reassuring participants about the safety and support procedures in place within the trial when recruiting people via remote methods. The public members also fed back their reflections on the findings, and one of the public members contributed to the trial publication as a co-author.

4: Discussion and conclusions: Comment on the extent to which PPI influenced the study overall. Describe positive and negative effects

Improving the acceptability of the research processes: Throughout the project, input from public members contributed to clearer and more plain language study materials. The public members also helped the researcher to reflect on the circumstances in which participants would receive this information, and the importance of conveying the correct meaning over using clinical terms (e.g. frailty, geriatrics). Their contributions to qualitative interview topic guides also ensured the approach to discussions with participants were more sensitive. In a few cases there were limits to changes that could be made: for example due to governance requirements for specific phrases in participant information sheets, or not being able to change text in validated questionnaires. However, such comments still helped pre-empt potential queries that participants may raise.

Broadening and enriching the interpretations of the findings: Public members' insights and suggestions

based in their lived experience relevant to COPD and frailty contributed to how the research team reflected on the findings. In many cases this was through direct feedback on findings, but sometimes other stories shared throughout project discussions helped keep the research grounded in the day-to-day experiences of people with COPD and frailty, and provided a source of information for ongoing reflections. For example, early discussions about the language used to talk about frailty continued to inform how the researcher discussed the project with potential trial participants. As such, their input not only broadened and enriched the interpretations of specific study findings, but also how the researcher understood and communicated about the project concepts.

Improve the clarity and impact of how the findings are shared with others: Public involvement throughout the project has improved the clarity of how the findings are shared. Involvement of a PPI member as a co-author helped to improve the clarity and sensitivity of academic outputs, and strengthened partnering plain-language outputs about frailty (e.g. an American Thoracic Society factsheet on frailty). We will continue to work with public members when sharing these findings.

5: Reflections/critical perspective: Comment critically on the study, reflecting on the things that went well and those that did not, so others can learn from this experience

Critical reflections on the strengths and learning for the future from this project are summarised below, in relation to the UK National Standards for Patient and Public Involvement in Research:

Standard	*Strengths*	*Learning for the future*
***Inclusive opportunities**:*	The project involved people with direct personal experience of COPD and frailty. Involvement was flexible and aligned to members' preferences and access requirements.	Representation in relation to protected characteristics was not known, and therefor diversity may have been lacking.
***Working together**:*	The researcher kept in touch in between meetings, recorded notes of contributions, and gave feedback following involvement to share the outcomes of their input.	Involvement opportunities were largely led by the researcher's requests. Collaborative planning of involvement at an earlier stage may have prompted more input.
***Support and learning**:*	The researcher gave an induction pack at the start, was responsive to queries, and provide additional support where requested.	The researcher could have proactively asked about learning interests and needs and offered more structured training.
***Communications**:*	Meeting materials were presented in plain language, and the researcher used communication methods in line with members' preferences (e.g. email, phone, post, in-person).	Not all members were comfortable with digital communications when in-person contacts were restricted due to Covid-19. Providing additional support for using technology may have helped communication.
Impact:	Public involvement has been reported within the study publications and shared as examples during training, teaching and presentations.	There is often insufficient space in publications to illustrate the full impact of involvement. The researcher could look to co-write a dedicated output about the impact of public involvement in this project to share this more widely.
Governance:	The researcher consistently reported back to public members throughout the project.	Study governance remained researcher led; more involvement here may have enhanced impact.

Supplementary Table S4: Missing item(s) in each clinical outcome measure by timepoint

**Scales**	**N participants with missing item(s)**			**Total % missing (n=85)**
	**Baseline (n=31)**	**90 days (n=27)**	**180 days (n=27)**	
Short Physical Performance Battery	20	27	27	87.1%
Chronic Respiratory Questionnaire				
Dyspnoea	1	6	5	14.1%
Fatigue	0	0	0	0%
Emotion	0	0	0	0%
Mastery	0	0	0	0%
Activities of Daily Living	1	0	3	4.7%
Euro-Qol 5D-5L				
Utility	1	1	3	5.9%
Visual Analogue Scale	1	1	3	5.9%
Hospital Anxiety and Depression Scale				
Anxiety	1	1	3	5.9%
Depression	2	1	3	5.9%
DeJong Gierveld Loneliness	1	1	3	7.1%

Supplementary Table S5: Scores on clinical outcome measures at each timepoint (median [IQR])

	**Usual Care**			**Comprehensive Geriatric Assessment + Usual Care**		
	**Baseline (n=16)**	**90-day (n=13)**	**180 day (n=12)**	**Baseline (n=15)**	**90-day (n=14)**	**180 day (n=15)**
Short Physical Performance Battery[a]	2.5 [1.8 - 5.8]	-	-	8 [4.0 - 10.5]	-	-
Chronic Respiratory Questionnaire						
Dyspnoea	2.5 [1.9 – 3.4]	3.2 [2.3 – 3.5]	2.9 [2.4 - 3.4]	2.4 [1.5 – 2.8]	2.8 [1.9 – 3.4]	3 [2.5 - 3.8]
Fatigue	3.0 [1.8 – 3.8]	3.8 [2.4 - 4.5]	2.6 [1.8 - 4.6]	2.5 [1.5 – 3.0]	2.5 [1.7 - 3]	2 [1.5 - 3.3]
Emotion	4.2 [3.2 – 5.4]	4.6 [3.9 - 5.5]	4.9 [3.6 - 5.6]	3.9 [3.0 – 4.3]	3.9 [2.8 - 5]	3.3 [2.6 - 5.1]
Mastery	4.3 [3.1 – 5.1]	4.8 [3 - 5.4]	4 [3.1 - 5.2]	4.3 [3.5 – 5.0]	4 [3 - 5.5]	4.8 [3 - 5.3]
Activities of Daily Living	11.5 [9.0 – 15.8]	11 [6 - 15]	12 [7 - 16]	13 [9.8 – 17.5]	14 [10.8 - 17]	12 [9 - 16.5]
Euro-Qol 5D-5L						
Utility	0.6 [0.4 – 0.8]	0.6 [0.5 - 0.7]	0.6 [0.5 - 0.8]	0.5 [0.4 – 0.7]	0.7 [0.4 - 0.8]	0.7 [0.2 - 0.7]
Visual Analogue Scale	52.5 [43.5 – 66.5]	50 [42.5 - 67.5]	45 [40 - 80]	49.5 [39.3 – 66.3]	55 [40 - 70]	70 [35 - 77.5]
Hospital Anxiety and Depression Scale						
Anxiety*	8.0 [2.8 – 12.8]	7 [4.5 - 8.5]	6 [2 - 8]	8.5 [2.8 – 12.3]	9 [2.5 - 11.5]	8 [4 - 10]
Depression*	7.5 [5.0 – 8.8]	8 [3 - 9.5]	7 [5 - 8]	8.0 [4.8 – 10.0]	6 [4 - 10.5]	5 [4 - 10]
DeJong Gierveld Loneliness*	2.0 [1.0 – 4.0]	1 [0.5 - 4.5]	1 [0 - 3]	3.0 [1.0 – 5.3]	3 [1 - 3]	2 [1 - 3.5]

*higher scores represent poorer function; [a]baseline n=6 control, n=5 intervention due to Covid-19 restrictions. IQR= Inter-Quartile Range

Supplementary Table S6: Service use at baseline and during the trial

Service use type	90 days prior to Baseline			0-180 days follow-up		
	**Total (n=31)**	**Control Group (n=16)**	**Intervention Group (n=15)**	**Total (n=27)**	**Control Group (n=12)**	**Intervention Group (n=15)**
Hospital						
Specialist outpatients	19	13	6	12	7	5
Day hospital	5	3	2	4	4	0
Hospital rehabilitation	1	1	0	1	1	0
Accident & Emergency	9	5	4	5	3	2
Inpatient stay	7	4	3	4	2	2
Critical care stay	1	1	0	0	0	0
Nursing/Residential stay	1	0	1	0	0	0
Hospice/Palliative Care	0	0	0	0	0	0
Other	5	3	2	4	1	3
Community						
GP (in-person)	14	8	6	11	5	6
GP (phone)	18	11	7	17	8	9
Other doctor at home	1	0	1	1	0	1
Social worker	1	0	1	0	0	0
Physiotherapist	2	2	0	2	1	1
Practice Nurse	13	9	4	11	8	3
District Nurse	0	0	0	2	0	2
Smoking cessation	3	3	0	1	1	0
Home Help	3	2	1	3	2	1
Optician	4	4	0	6	3	3
Dentist	1	1	0	4	2	2
Chiropodist / Podiatrist	6	3	3	5	2	3
Psychologist	1	1	0	0	0	0
Dietician	0	0	0	4	1	3
Hospice / Palliative care	0	0	0	0	0	0
Other	4	1	3	8	3	5
Tests						
Respiratory function	10	6	4	7	4	3
Chest X-ray	9	6	3	11	6	5
Echocardiogram	3	3	0	6	1	5
Electrocardiogram	5	5	0	6	3	3
Blood gas	6	2	4	4	1	3
MRI Scan	5	3	2	4	0	4
CT/CAT Scan	11	8	3	8	4	4
Blood test	23	13	10	20	8	12
Other	9	7	2	7	2	5
Equipment						
Ambulatory oxygen	2	1	1	3	1	2
Long-term oxygen therapy	3	2	1	2	2	0
Non-invasive ventilation	1	1	0	3	2	1
Occupational aids	17	11	6	13	7	6
Unpaid/family Care						
Personal care	11	7	4	8	5	3
Medical procedures	8	7	1	6	5	1
Help at home	19	13	6	14	7	7
Help outside home	18	10	8	21	10	11
Time 'on call'	7	5	2	10	6	4
Other	9	5	4	3	2	1
Missing service use data	1	0	1	3	1	2

GP = General Practice. Note: Examples of 'other' for: hospital care= vaccine for Covid-19, physiotherapy assessment; Community care= chair exercise, paramedic visit; Tests= Cystoscopy, Endoscopy; Unpaid/family Care = managing garden, attending social care appointment

Supplementary Table S7: Qualitative interview purposive sampling for participants (n=7)

Characteristic	n
Site	
A	4
B	3
Intervention fidelity	
No CGA actions completed	2
Some CGA actions completed	2
All CGA actions completed	3
Living status	
Alone	2
With others	5
Outcomes (Chronic Respiratory Questionnaire)[a]	
Improved	2
Worsened	2
Mixed	3
Questionnaire completion	
Missing ≥ 1 timepoint	0
All timepoints complete	7

CGA = Comprehensive Geriatric Assessment, [a]Observed changes across Chronic Respiratory Questionnaire domains between baseline and 90-day measures (Improved = scores increased across ≥3 domains, Worsened = scores decreased across ≥3 domains, Mixed = all other outcomes).

Supplementary Table S8: Qualitative interview purposive sampling for professionals (n=5)

**Characteristic**	**n**
Site	
A	2
B	3
Discipline	
Pulmonary rehabilitation	3
Geriatrics	2
